# Spatiotemporal distribution of acquired immunodeficiency syndrome incidence in Brazil between 2012 and 2016

**DOI:** 10.1590/0037-8682-0086-2019

**Published:** 2019-12-20

**Authors:** Edson Zangiacomi Martinez, Miriane Lucindo Zucoloto, Guilherme Galdino, Altacílio Aparecido Nunes, Elisangela Aparecida da Silva Lizzi

**Affiliations:** 1 Universidade de São Paulo, Faculdade de Medicina de Ribeirão Preto, Ribeirão Preto, SP, Brasil.; 2 Universidade de Ribeirão Preto, Ribeirão Preto, SP, Brasil.; 3 Universidade Tecnológica Federal do Paraná, Cornélio Procópio, PR, Brasil.

**Keywords:** AIDS, Epidemiology, Spatial modeling

## Abstract

**INTRODUCTION:**

Acquired immunodeficiency syndrome (AIDS) remains a major public health issue in Brazil. This ecological study aimed to evaluate the spatiotemporal distribution of notified new AIDS cases in Brazil between 2012 and 2016.

**METHODS:**

A Bayesian spatiotemporal model based on the Poisson distribution was used to obtain smoothed incidence estimates of AIDS in each of the 133 Brazilian intermediate regions.

**RESULTS:**

Spatial distribution of new AIDS cases is highly heterogeneous. Regions with higher gross domestic product per capita tend to have higher incidence rates of AIDS.

**CONCLUSIONS:**

Strategies to prevent and control AIDS should consider regional differences.

An ecological study aiming to describe the burden of acquired immunodeficiency syndrome (AIDS) and its trends in Brazil from 1980 to 2015 showed that the incidence of this disease remains high, with a tendency to increase in the incoming years^1^. Additionally, it is estimated that currently in Brazil, approximately one in every five men who have sex with men is infected with human immunodeficiency virus (HIV)^2^. Considering this scenario, it is imperative that efforts must be made to improve the performance of AIDS prevention programs, taking into account the significant heterogeneity and complexity of the distribution of the disease in Brazil due to cultural, sociopolitical, and economic factors of the different regions^3^.

Considering the 27 Brazilian Federal Units as units of analysis, an ecological study evaluated the spatiotemporal distribution of the standardized incidence rates of AIDS among adults in Brazil from 2006 to 2012 and showed significant regional differences in the disease incidence^4^. The authors showed that Federal Units with higher Human Development Index (HDI) had higher incidence of the disease than Federal Units with lower HDI^4^. Similarly, a study on the geographic distribution of AIDS in the State of Rio de Janeiro, Southeast Brazil, showed a heterogeneous distribution pattern of the disease across the municipalities of the state and a significant association between the number of new cases of the disease and socioeconomic variables such as income inequality, income per capita, and the education component of HDI^5^. Consequently, considering the administrative districts of São Paulo City as units of analysis, another ecological study^6^ showed a positive association between HDI and AIDS incidence among males from 2000 to 2010, but no associations were observed between these variables from 2011 to 2016. These studies are important since they provide a better understanding on the association between AIDS incidence and surrounding socioeconomic conditions and how geography influences these associations^6^, and their results are useful for public health planners and policy-makers in designing more effective preventive strategies and services.

In the present study, we used spatial statistical methods to describe the spatiotemporal distribution of notified new AIDS cases in Brazil between 2012 and 2016, considering the intermediate regions as units of analysis. In 2017, the regional geographic division of Brazil was based on clusters of municipalities called mesoregions and microregions. This year, the Brazilian Institute of Geography and Statistics (IBGE) proposed a new division, where the municipalities are now clustered into intermediate and immediate geographic regions^7^. Immediate regions are groups of adjacent municipalities that are clustered together taking into account their geographic features and have a local urban center as a base. Intermediate regions are groupings of immediate regions that are articulated by the influence of one or more metropolitan regions, regional capitals, and/or representative urban centers. In this context, data from the Internet free access Brazilian HealthCare Computer System (DATASUS, Departamento de Informática do SUS) regarding the number of new AIDS cases in each municipality were collected and subsequently clustered accordingly to the corresponding intermediate regions. The study also investigated the association between the gross domestic product (GDP) per capita of the intermediate regions and the AIDS incidence in 2016. Data on GDP was obtained from the IBGE.

The Global Moran’s Index (I^2^) was estimated annually to detect spatial heterogeneity in the distribution of the disease, and Local Indicators of Spatial Association (LISA) were used to identify the locations of significant clusters of AIDS incidence. A Bayesian spatiotemporal model^8^ was used to obtain smoothed incidence estimates of AIDS in each of the R = 133 intermediate regions. It was assumed that Y(p,s,t), the number of AIDS cases notified in the intermediate region p (p=1,…,133), considering gender s (s=1 for males, s=2 for females) and year t (t=1 for 2012, t=2 for 2013, and so on), followed a Poisson distribution with mean given by N(p,s,t) × m(p,s,t). In this case, N(p,s,t) was the population for the intermediate region p, considering gender s and year t (data from IBGE), and m(p,s,t) was the correspondent incidence rate given by m(p,s,t) = exp[a(s,t) × w(s,p) × b(p,s,t)]. In the hierarchical Bayesian analysis, a(s,t) were unknown parameters following a s-variate normal distribution with zero means and a covariance matrix following a Wishart distribution, and b(p,s,t) were random interaction terms following a normal distribution with different variances annually. Additionally, it was assumed that the spatial effects w(s,p) followed a bivariate normal intrinsic conditional auto-regressive structure. A similar model, including the effect of a covariate, was used to investigate the association between the GDP per capita of the intermediate regions and the AIDS incidence. R software was used to obtain the values of Moran and LISA statistics, and OpenBUGS software was used to fit the Bayesian model to the data, based on Markov chain Monte Carlo methods^8^. All these statistical methods were based on Queen spatial weights.

Figure 1 shows the spatial distribution of the smoothed incidence rates of AIDS for males and females, considering the years 2012, 2014, and 2016. Maps for the other years are not shown because of the large number of data. Considering the male population, in 2012, the median smoothed AIDS incidence was 17.68 cases per 100,000 inhabitants among the 133 intermediate regions (interquartile range [IQR], 10.99 to 27.15). In 2014, this median incidence was 19.42 cases per 100,000 inhabitants (IQR, 12.25 to 28.60), and in 2016, the median incidence was 18.18 cases per 100,000 inhabitants (IQR, 11.55 to 26.91). In 2012, the Brazilian intermediate regions had a median smoothed incidence of 11.19 cases per 100,000 inhabitants among females (IQR, 7.36 to 16.15). In 2014, this median incidence was 10.37 cases per 100,000 inhabitants (IQR, 6.83 to 14.94), and in 2016, the median incidence was 8.44 cases per 100,000 inhabitants (IQR, 5.75 to 12.88).

**FIGURE 1:**
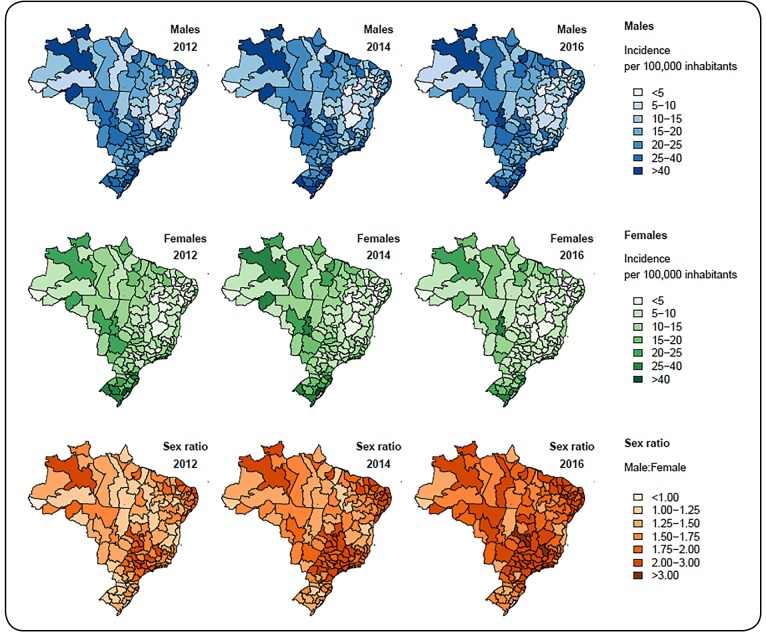
Spatial distribution of the smoothed acquired immunodeficiency syndrome (AIDS) incidence rates among males (first line) and females (second line) obtained from the spatiotemporal model. Spatial distribution of the male to female AIDS incidence ratio (third line).

After smoothing, the highest AIDS incidence rates per 100,000 inhabitants among males in 2016 were 71.04 cases in the intermediate region of Florianópolis (State of Santa Catarina, South Region), 59.14 cases in Manaus (North Region), 56.4 cases in Porto Alegre (State of Rio Grande do Sul, South Region), 51.88 cases in Boa Vista (North Region), 51.83 cases in Belém (North Region), and 45.98 cases in Rondonópolis (Center-West Region). In females, the highest rates per 100,000 inhabitants in 2016 were 36.31 cases in the intermediate region of Porto Alegre, 35.09 cases in Florianópolis, 26.42 cases in Pelotas (State of Rio Grande do Sul), 45.98 cases in Rondonópolis, 51.83 cases in Belém, and 42.89 cases in Blumenau (State of Santa Catarina).

Figure 1 also shows the maps describing the spatial distribution of the male to female incidence ratio (IR) for AIDS in the Brazilian intermediate regions. In the years 2012, 2014, and 2016, the median for the male to female IR, were 1.51 (IQR, 1.27 to 1.78), 1.73 (IQR, 1.49 to 2.05), and 1.98 (IQR, 1.73 to 2.36), respectively. The results suggest a deceleration in the feminization of the disease, since the male to female IR between 1980 and 1990 was 6.5:1, and in the period between 1991 and 2001, it was 2.4:1^9^.

After smoothing, in 2016, the highest male to female IR was 4.65 reported cases among males for every 1 reported case among females in the Federal District (where Brasilia is located, the Federal Capital). There is an expressive difference between this value and the others, observed in descending order: 3.70 in Belo Horizonte (State of Minas Gerais, Southeast Region), 3.29 in São Paulo (State of São Paulo, Southeast Region), 3.18 in Goiânia (State of Goiás, Center-West Region), 3.13 in Juazeiro do Norte (State of Ceará, Northeast Region), and 3.06 in João Pessoa (State of Paraíba, Northeast Region). In other regions, the correspondent male to female ratios were lower than 3. With the exception of Juazeiro do Norte, all these intermediate regions are located in the corresponding state capitals, which are large urban centers with high population density.

Figure 2 shows LISA maps generated for 2012, 2014, and 2016. This figure is used to identify spatial clusters and examine the spatiotemporal patterns of the disease incidence. Red colors indicate intermediate regions with high IR of AIDS whose neighbors have high incidence rates (high-high spatial correlation), and blue colors indicate intermediate regions with low incidence rates of AIDS whose neighbors have low incidence rates (low-low spatial correlation). During the study period, the values of the Moran’s Index (I^2^) confirmed that the smoothed incidence rates of AIDS for males and females and the male to female IR for AIDS are spatially heterogeneously distributed across the Brazilian intermediate regions (Figure 2, p values <0.01).

Figure 1 shows that, considering both male and female populations, the number of AIDS cases reported in the Northeast Region of Brazil tends to be higher in the coastal areas, where the most populous cities are located, than in the noncoastal areas. Additionally, maps in the first and second rows of Figure 2 show a large low-low cluster in the Northeast Region, not including the coastal areas. In contrast, these maps also show a high-high cluster in the South Region, including mainly the coastal regions of the States of Santa Catarina and Rio Grande do Sul, where there is a greater flow of people and goods. According to the study by Silva-Lizzi et al.^4^, higher incidence rates in these Brazilian states in 2006-2012 were also observed. These authors observed that the subtype C of HIV-1 and C-containing sequences are highly prevalent in Southern region, mainly in Santa Catarina and Rio Grande do Sul states, and it is reported that this subtype seems to spread faster than other subtypes of group M^4^. Additionally, Pereira et al.^10^ described the burden of AIDS in Rio Grande do Sul as a result of the low state investment in actions to prevent and control sexually transmitted infections.

**FIGURE 2:**
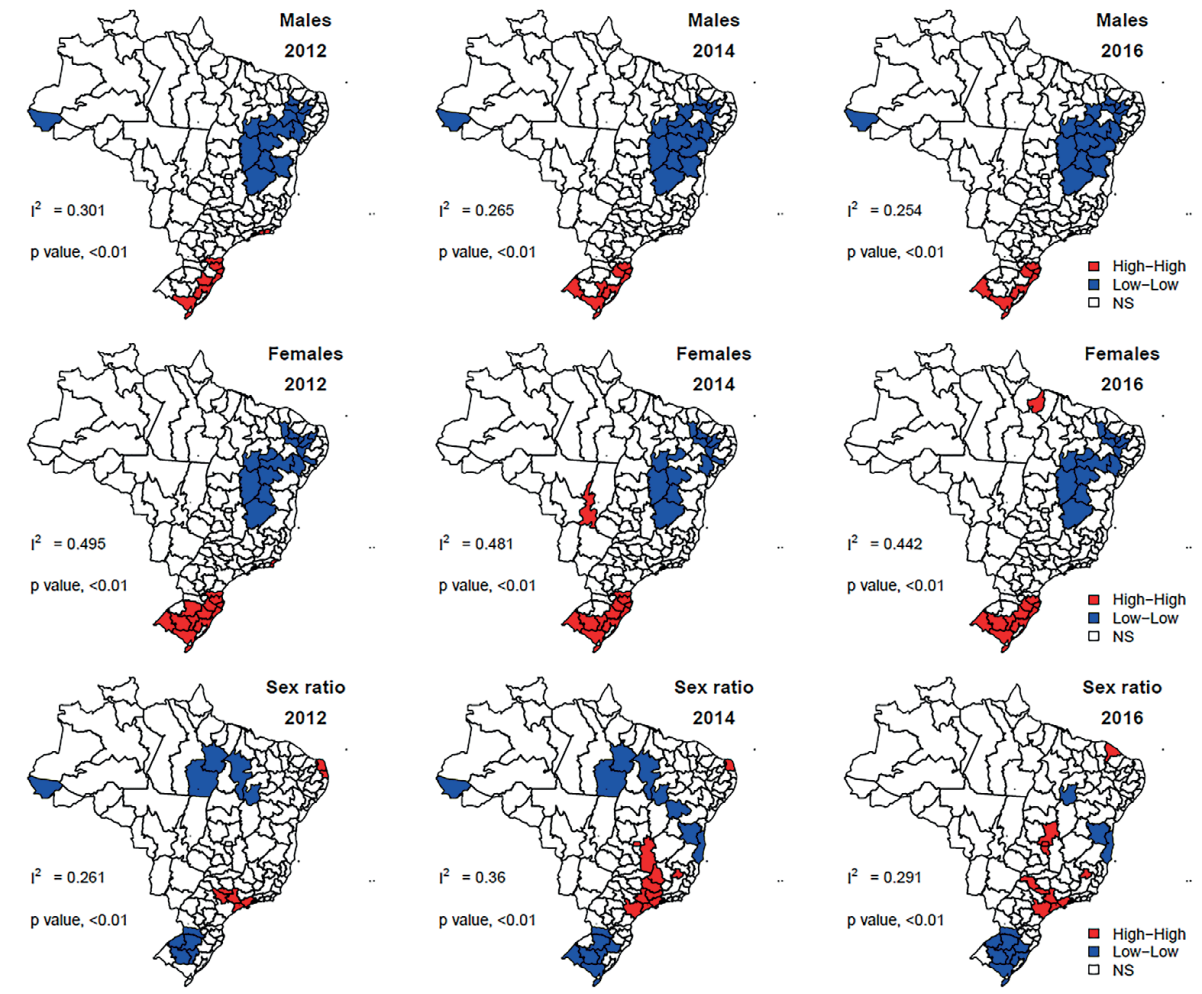
Global Moran’s Index (I^2^) and Local Indicators of Spatial Association maps of the smoothed acquired immunodeficiency syndrome (AIDS) incidence rates among males (first line), females (second line), and the male to female AIDS incidence ratio (third line).

The graphs in Figure 3 show the association between GDP per capita and the number of new AIDS cases notified in 2016 among males and females. The trend lines in the two graphs were obtained from a Bayesian regression model, and the 95% credible interval (95% CI) for the parameters related to the association between these variables did not include the zero value, suggesting significant associations (1.96 among males, 95% CI, 1.53-2.37; 1.67 among females, 95% CI, 1.22-2.12). These results indicate that the intermediate regions with higher GDP per capita tend to have higher incidence rates of AIDS than intermediate regions with lower GDP per capita. However, it is important to remember that the disease is also present in the North and Northeast regions, which are considered less developed than the Southeast and South Regions.

**FIGURE 3:**
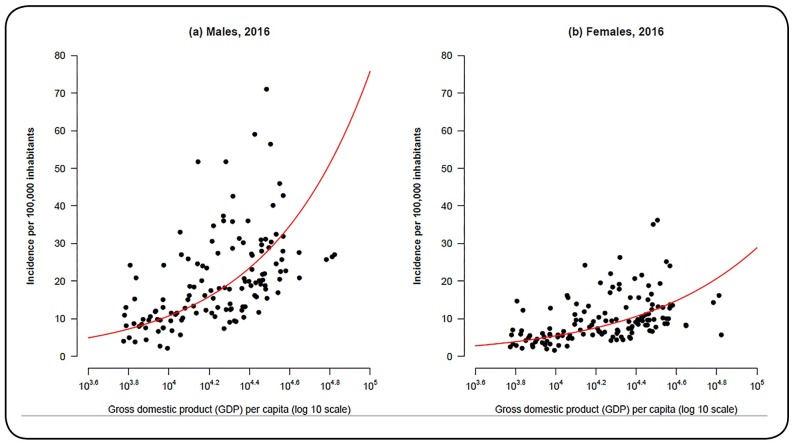
Association between gross domestic product per capita (log 10 scale) and acquired immunodeficiency syndrome incidence notified in 2016 among **(a)** males and **(b)** females.

Ecological studies are important in the identification of high-risk areas for a disease. While the growing AIDS incidence in Brazil^1^ requires increasing investments in disease prevention and treatment programs, the significant variability in HIV epidemic growth patterns among the Brazilian regions^4^ ^,^ ^11^ ^,^ ^12^ requires new studies that characterize the disease in different regions from the country according not only to its clinical and epidemiological profile but also to its social and cultural aspects.

Interesting results, such as the greater occurrence of the disease in regions with the highest GDP per capita, point to a reality that is slightly different from that found in other regions of the world, such as Asia^13^, where AIDS is consistently growing among the impoverished population, with a lower GDP per capita, a phenomenon known as “pauperization of the epidemic,” which was registered in Brazil in the late 1990s and the early 2000s. This change reflects the risk behavior and the forms of transmission of HIV^14^ ^,^ ^15^.

A potential limitation of this study is the quality and completeness of information on the Brazilian AIDS cases database, which is subject to delays and underreporting. The dataset used in this study is only available at the aggregate level. However, the obtained results may be useful as an update of the results of similar studies^4^ ^,^ ^11^ ^,^ ^12^ and reinforce the constant need for further studies on disease surveillance in Brazil.
